# Mosquito Surveillance and Insecticide Resistance Monitoring Conducted by the Florida Keys Mosquito Control District, Monroe County, Florida, USA

**DOI:** 10.3390/insects13100927

**Published:** 2022-10-13

**Authors:** Lawrence J. Hribar, Michael B. Boehmler, Heidi L. Murray, Catherine A. Pruszynski, Andrea L. Leal

**Affiliations:** 1 Florida Keys Mosquito Control District, Marathon, FL 33030, USA; 2 Florida Keys Mosquito Control District, Key Largo, FL 33037, USA; 3 Florida Keys Mosquito Control District, Key West, FL 33040, USA

**Keywords:** Florida Keys, light trap, BG Sentinel, oviposition trap, adulticide, larvicide, bottle bioassay, mosquito-borne disease, integrated pest management

## Abstract

**Simple Summary:**

Mosquitoes are the most important group of flies affecting human health and wellness. Worldwide, mosquito-borne diseases kill over 700,000 people every year and afflict millions more. Mosquitoes also adversely affect domestic and agricultural animal health and negatively impact tourism-based economies. The Florida Keys Mosquito Control District is responsible for reducing mosquito populations in an area stretching from Cross Key and Broad Creek in the northeast to Key West in the southwest. Before mosquitoes can be controlled, it is important to know how many mosquitoes there are, what species are present, and where they are located. Adult mosquito surveillance traps such as the BG Sentinel, set around homes and businesses, and light traps, set in field sites, are used to identify the number and species of mosquitoes in an area. Larval and pupal mosquito surveillance is conducted by visiting water sources in domestic and field habitats to look for immature mosquitoes. Surveillance informs operational staff of the types of treatments required to control mosquitoes present. Products currently used to kill larval and adult forms of mosquitoes are continuously evaluated for efficacy to reduce pesticide-resistant populations.

**Abstract:**

Mosquito control programs in the State of Florida are charged with protecting human and animal health, fostering economic development of the State, permitting enjoyment of the natural attractions in Florida, and improving the quality of life of citizens. Mosquito control programs must accomplish these tasks in such a manner as will protect the environment and terrestrial, marine, and freshwater ecosystems. The Florida Keys Mosquito Control District provides a science-based Integrated Pest Management mosquito control program to the residents of the Florida Keys, Monroe County, Florida. Operational decisions are based on surveillance of adult and immature mosquitoes. Mosquito populations are monitored by means of carbon dioxide-baited light traps BG Sentinel traps, truck traps, gravid traps, oviposition traps, and human landing rate counts. Larvae and pupae are monitored by inspections of natural and human-made immature habitats. Due to past and current reliance on chemical pesticides for control of mosquitoes, the District maintains a pesticide resistance detection program consisting of CDC bottle bioassays and larval bioassays, challenging local mosquito species with currently used adulticides and larvicides.

## 1. Introduction

For millennia, mosquitoes have played an important role in human lives due to their capacity as vectors. Mosquitoes transmit pathogens that cause diseases such as malaria, dengue, chikungunya, West Nile fever, yellow fever, and Zika. There are millions of cases of vector-borne disease recorded each year, resulting in 700,000 deaths [1]. Within the last decade, Florida and the Florida Keys have seen human cases of multiple mosquito-borne diseases including dengue, Zika, and West Nile fever, all of which occurred in South Florida [2,3,4]. Of the 3698 mosquito species found throughout the world, only some of them are vectors of disease [5]. Focusing on the surveillance of vector species allows mosquito control operations to target the species of most concern in their area of responsibility. Monitoring these vectors can assist in identifying at-risk areas, population abatement, and ultimately the reduction of disease transmission [6]. In order to successfully intervene between humans and mosquito vectors, mosquito control agencies employ a system of integrated tactics known as integrated pest management (IPM).

An IPM program uses a science-based multidisciplinary approach to control all mosquito life stages [7]. These approaches include surveillance and identification, source reduction, control through larvicides and adulticides, and public outreach and education. Surveillance allows for appropriate control decisions based on action thresholds and species present. Source reduction eliminates larval mosquito habitats and can be very effective in the reduction of mosquito populations. This can include simple tasks such as discarding or overturning water-holding containers or large-scale efforts such as ditching to enhance water flow and creating impoundments. When source reduction cannot occur, treating larval habitats with available larvicides is the next step in mosquito control. There are a variety of materials currently classified as larvicides, including insect growth regulators, microbial larvicides, organophosphates, and surface oils and films [8]. Adulticiding is often considered the method of last resort in an IPM program [9]. Adulticides are broad-spectrum pesticides utilized for the control of adult mosquitoes. There are currently only two registered classes of adulticides for use in Florida, organophosphates and pyrethroids [9]. Due to the limited number of active ingredients in use throughout mosquito control, resistance testing is essential to ensure control levels are achieved for both larval and adult treatments. Public education is also an important aspect of an IPM program because it can lead to source reduction, reducing the need for treatment.

Following closely to an IPM program corresponds well with guidelines set by the legislative bodies governing mosquito control agencies. The State of Florida has had organized mosquito control since 1922 [10]. Mosquito control programs were created to maintain such levels of arthropod control as will protect human health and safety and foster the quality of life of the people, promote the economic development of the state, and facilitate the enjoyment of its natural attractions by reducing the number of pestiferous and disease-carrying arthropods [11]. Mosquito control programs are governed by the Florida Department of Agriculture and Consumer Services (FDACS) which oversees individual programs, including adherence to best mosquito practices and treatments action thresholds stated in Florida law. Florida Chapter 5E-13.036 states that applications of adulticides can only occur with a quantifiable increase in population, when populations exceed 25 mosquitoes per trap per night, or requests for service are confirmed by standard surveillance methods [12]. Additionally, aerial adulticide applications are justified along beaches and bay shores when there is a demonstrable three-fold increase over an established base population [12]. All programs must keep surveillance records for three years in the State of Florida [12].

The Florida Keys are part of the South Florida rockland habitat ecosystem divided into two groups of islands according to the bedrock. The Upper Keys (Soldier Key in Miami-Dade County to Big Pine Key in Monroe County) are underlain by Key Largo Limestone; the Lower Keys (Big Pine Key to Key West) are underlain by Miami Limestone. Most of the islands are low elevation, 1 to 2 m above sea level. Dominant vegetation in the Florida Keys is tropical hammocks and pinelands [13]. The Upper Keys and Lower Keys can differ in terms of vegetation, average temperature, rainfall, and permeability of bedrock [13,14,15,16]. The Florida Keys are in a transition zone between Nearctic and Neotropical biological realms; therefore, the mosquito fauna is indicative of this transition zone [17]. All of these conditions create differing mosquito populations and abundance throughout the Keys and emphasize the importance of surveillance.

**Figure 1:**
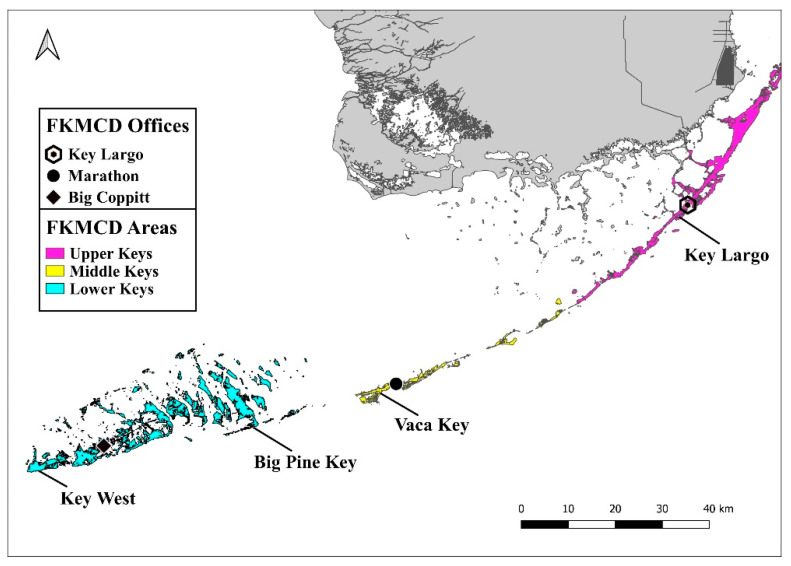
Locations of FKMCD offices, operational areas within the Florida Keys, and localities mentioned in the text.

The Florida Keys Mosquito Control District (FKMCD), originally known as the Monroe County Anti-Mosquito District, was established in 1949. FKMCD is an independent taxing district with approximately seventy full-time employees and a current operating budget of approximately USD15 million. The mission of FKMCD is to protect the public from health threats and nuisance issues that impact the local economy by utilizing control methods that are efficient, effective and environmentally sensitive. There are three District offices evenly spaced throughout the Keys. The Big Coppitt Key office provides ground operations for the Lower Keys (Key West through the Seven Mile Bridge), the Vaca Key office provides ground operations for the Middle Keys (Seven Mile Bridge through Lower Matecumbe Key) and houses an aerial fleet used to apply larvicides and adulticides throughout the Keys, and the Key Largo office houses ground operations for the Upper Keys (Upper Matecumbe Key through Key Largo) (Figure 1). The IPM program employed by FKMCD includes field inspectors conducting landing rate counts, public education, source reduction, applying larvicides and adulticides; aerial operations performing larvicide and adulticide applications; and research personnel conducting field studies, surveillance, and resistance testing. Nuisance mosquitoes were of primary concern for FKMCD for many years; however, with the reintroduction of dengue to the Florida Keys in 1999, emphasis has also been placed on the control of vector species [2].

Surveillance of larval and adult mosquitoes is an important aspect for all control operations. Surveillance can be comprised of many different methods, all resulting in a better understanding of population abundance, seasonality, species composition, and resistance monitoring [18]. This review reports on the methods of surveillance used by the FKMCD. These surveillance activities include adult surveillance by carbon dioxide-baited light traps, BG Sentinel traps, truck traps, gravid traps, oviposition traps, and landing rate counts, larval and pupal surveillance, and insecticide resistance monitoring of larvicides and adulticides (Table 1). Each method has its own niche in mosquito control, from establishing action thresholds to monitoring insecticide resistance, all of which are important for control programs.

## 2. Adult Surveillance

Regular, repeated, and ongoing mosquito surveillance is foundational to mosquito control programs [19]. Mosquito surveillance allows the agency to detect changes in the seasonal distribution, relative abundance, and species composition of mosquito populations and thus allows the control program to get a head start on controlling both nuisance mosquitoes and disease vectors [20]. Collecting high-quality surveillance data is critical during outbreaks of vector-borne disease, both to prevent the spread of disease and for effective control of the vector species [21]. The FKMCD has used a number of surveillance methods depending on the species of interest and the reason for conducting surveillance.

### 2.1. Carbon Dioxide-Baited Light Traps

The American Biophysics Company (ABC) trap (available from Clarke, Roselle, IL, USA) and the John W. Hock trap (Gainesville, FL, USA) are modifications of the original Centers for Disease Control (CDC) light trap developed by Sudia and Chamberlain [22]. The addition of an insulated chamber to hold solid carbon dioxide (dry ice) increases the catch over the use of a light source alone [23]. A century ago, carbon dioxide was found to attract mosquitoes [24]. The attraction of hematophagous Diptera to carbon dioxide depends on a plume dispersing downwind at levels above background concentration [25]. Results obtained from carbon dioxide-baited traps should be interpreted with some caution because even the best traps may collect less than 60% of the mosquitoes attracted by the plume [26]. FKMCD has used carbon dioxide-baited light traps for mosquito surveillance since 1998 [27]. Currently, FKMCD sets 62 ABC and CDC CO_2_-baited light traps throughout the Florida Keys on a weekly basis. The light source is not used ubiquitously throughout the Florida Keys. Traps are placed in the late afternoon and retrieved the following morning. The number of mosquitoes captured varies considerably. Collections of zero mosquitoes are uncommon but not unknown; usually, this is due to windy conditions. The largest number of mosquitoes collected in one trap during a single trap night was 446, 467 *Aedes taeniorhynchus* Wiedemann from an ABC trap set on Little Pine Key on 19 August 2002.

ABC traps baited with carbon dioxide have been used in all of the surveillance studies conducted by FKMCD. These collections have resulted in new distribution records or confirmation of old collection records for *Aedeomyia squamipennis* (Lynch Arribalzaga), *Aedes albopictus* Skuse, *Aedes bahamensis* Berlin, *Aedes condolescens* Dyar and Knab, *Aedes pertinax* Grabham, *Aedes scapularis* Rondani, *Anopheles albimanus* Wiedemann, *Anopheles grabhamii* (Theobald), *Psorophora johnstonii* (Grabham), *Culex coronator* Dyar and Knab, *Culex declarator* Dyar and Knab, *Culex peccator* Dyar and Knab, *Culex tarsalis* Coquillett, and *Culiseta inornata* (Williston) [28,29,30,31,32,33,34,35,36,37,38,39,40].

Data and specimens from surveillance traps, if collected over a long enough period of time and in a stable collection site, can provide insight into seasonality, relation to climatic conditions, and vector status. Seasonal distribution data for mosquitoes from distinct islands were used to show that every island has its own particular mosquito fauna [27,41,42,43,44,45,46]. The uncommon species *P*. *johnstonii* was found on several islands within the Florida Keys and on No Name Key it was present only episodically, with periods of two to four years between large emergences [47,48].

Mosquito surveillance data over a period of nearly twenty years revealed gradual declines in mosquito numbers on Big Pine Key, No Name Key, and Vaca Key [49,50]. On Big Pine Key and No Name Key a reduction of adult mosquito numbers correlated with increased larval control activities on neighboring islands [49]. Number of aerial adulticide missions flown per year and hectares treated with aerially applied adulticide was reduced significantly on Big Pine and No Name Keys following the initiation of larviciding on nearby uninhabited islands [49]. Migration of *A. taeniorhynchus* between islands was demonstrated via a mark-recapture study, wherein it was discovered that mosquitoes emerging from different uninhabited islands migrated to particular populated islands [51].

Mosquitoes collected during routine surveillance were sent to other laboratories to generate new information on the vector status of local mosquitoes relative to arboviruses. Seven species were found naturally infected with West Nile Virus: *Anopheles atropos* Dyar and Knab, *A. condolescens*, *A. taeniorhynchus*, *Culex erraticus* Dyar and Knab, *Culex nigripalpus* Theobald, *Culex quinquefasciatus* Say, and *Deinocerites cancer* Theobald [52,53]. *Aedes taeniorhynchus* and *Cx. erraticus* were found to be relatively efficient vectors of Rift Valley Fever Virus [54].

Data from mosquito surveillance were analyzed in conjunction with meteorological data to relate mosquito trap collections with environmental conditions, wind speed, wind direction, and temperature were found to be important variables [55,56,57].

Four data papers containing data from multiple years of trapping on different islands have been published, allowing other researchers to access and use those data from Cross Key, Grassy Key, Long Key, and Vaca Key [58,59,60,61]. It is worth noting that examination of mosquito surveillance bycatch has resulted in numerous new distributional records for other arthropod species [62].

### 2.2. BG Sentinel Traps

In August 2009, the first autochthonous case of dengue in Florida in 70 years was diagnosed in a New York City resident who had vacationed in Old Town; a neighborhood in Key West. This discovery prompted an immediate response from the FKMCD, charged with surveillance and abatement of the vector, *Aedes aegypti* Linnaeus. Along with the treatment of properties with confirmed or suspected dengue cases, FKMCD deployed BG Sentinel traps to survey the *A. aegypti* population.

The BG Sentinel trap (Biogents, Regensberg, Germany) was designed to attract host-seeking mosquitoes using a proprietary human-scented lure, convection currents that mimic human movement, and contrasting visual components [63]. The trap consists of a collapsible, blue fabric base 40 cm tall and 36 cm in diameter and a round white perforated plastic top with a black funnel opening in the center. The trap requires a 12-volt battery to run the small but powerful 3.6-watt ventilator. While the BG Sentinel is designed to collect host-seeking mosquitoes, non-targets, and predators such as ants and small reptiles can also be attracted to the mosquitoes attracted to the trap [64,65]. Based on a multitude of trap comparison and sampling studies that demonstrated the BG Sentinel trap’s efficacy collecting *Aedes* (*Stegomyia*) mosquitoes [66,67,68,69,70,71], the District began using BG Sentinel traps in Old Town to better evaluate the vector population of *A. aegypti*.

The BG Sentinel traps were set in a combination of locations that included confirmed positive and suspected dengue cases as well as known *A. aegypti* “hot spots” (e.g., properties requiring repeat inspections due to recrudescent larval habitats). Traps were set overnight once per week for approximately 20 h. In addition to the BG-Lure and octenol, 1.5 kg of dry ice was also added as an attractant to collect more host-seeking female *A. aegypti* [70].

Immediately after FKMCD was notified of that first reported dengue case, twenty-eight BG Sentinel traps were set weekly. Over the course of the outbreak that lasted through 2010, between 30 and 40 traps were deployed weekly in Key West, requiring a full-time trap technician. Mosquitoes collected from each trapping location were identified by a certified mosquito identification specialist and *A. aegypti* females were placed into vials and sent to the Florida Department of Health or Florida Gulf Coast University (FGCU) for virus testing. In a note published by Graham et al. [72], three out of 1178 mosquito pools sent to FGCU tested positive for dengue virus serotype 1.

The established Key West BG Sentinel traps were not only used for *A. aegypti* surveillance, but to assist in measuring mosquito population changes over time. Abundance data were instrumental in determining effectiveness of new abatement methods such as the area-wide application of liquid *Bacillus thuringiensis israelensis* Barjac (Bacillales: Bacillaceae) (Bti) developed by FKMCD and Valent Bioscience Corporation [73]. The BG Sentinel trap data were also used in a lethal ovitrap trial [74] and a small-scale release of *Wolbachia*-infected *A. aegypti* males [75]. Individual traps were set when FKMCD inspectors were unsure of which mosquito species were responsible for a service request as BG Sentinels can also attract *C. quinquefasciatus* [76,77]. The BG Sentinel traps are also adept at collecting *A. aegypti* in a range of physiological states [78], so in another study, blood-engorged *A. aegypti* and *Cx*. *quinquefasciatus* were captured in BG Sentinel traps in Key West, Marathon, and Key Largo and blood meals were identified. Knowledge of blood meal preferences in these areas led to a better understanding of the ecological factors influencing dengue transmission in the Keys [79]. Murray et al. [36] also reported the use of BG Sentinel traps in collections of the invasive mosquito, *A. albopictus*, which prior to 1993 had not been seen in the Florida Keys.

Over time, the number of BG Sentinel traps set weekly in Key West was reduced to a more manageable 14 traps. Relative population data changes inform operations with the creation of an action threshold of average *A. aegypti* females collected per trap night. This threshold was developed by evaluating the average female *A. aegypti* collected per trap night from each trap location during the 2010 outbreak and compared to subsequent years. These data are available as open access for future statistical analysis and modeling [80].

After initiating *A. aegypti* surveillance in Key West with BG Sentinel traps, FKMCD wanted to consider the extent of the dengue vector issue throughout the island chain. In the Upper Keys, initial trapping locations were chosen based on historic reports of high abundance of *A. aegypti* in both adult and larval inspections [81]. Four trapping locations were chosen in 2010, and BG Sentinels are still set weekly as of this writing.

In the Middle Keys city of Marathon, besides routine surveillance, BG Sentinels were an integral part of the Zika surveillance response in 2016. While there were no reported locally acquired cases of Zika transmission in the Florida Keys, there were 191 autochthonous cases reported in Miami-Dade County, a county bordering Monroe [82]. As infection with Zika virus can have serious health effects on fetuses [83], FKMCD created a Zika pregnancy registry to assist protecting this vulnerable population. Pregnant residents and visitors alike could add their name to the registry and extra attention would be made at their lodgings to ensure low presence of *A. aegypti*. This extra attention included inspections and regular setting of BG Sentinels. The registry expanded into all of Monroe County, but no additional names have been added to the registry since 2019.

BG Sentinels have been used to measure objectives for innovative vector control methods like the Oxitec genetically modified *A. aegypti* male release, a collaboration between Oxitec LTD and FKMCD taking place in Marathon [84]. As the trial is still ongoing, more information on collections will be available later.

In January 2020, the first locally acquired case of dengue was reported in Key Largo. FKMCD immediately implemented the emergency response plan for *A. aegypti* control which included additional vector surveillance. Over the next four months, thirteen BG Sentinel traps were set in suspected and confirmed case locations throughout the Upper Keys. Female *A. aegypti* collected from the traps were sent to Centers for Disease Control in Puerto Rico and tested for dengue. Results yielded three positive pools out of 1330 mosquitoes and all three were dengue serotype 1 (FKMCD, internal communication). The last human case reported for the outbreak was in August 2020, and no additional cases have been reported in the Upper Keys as of this writing. As was the case in Key West in 2010, many of the dengue traps have become a permanent part of *A. aegypti* surveillance in the Upper Keys.

The BG Sentinel trap has enabled FKMCD to specifically collect for *A. aegypti*, but its utility has not been limited to surveillance only. Thanks to this innovative trap, FKMCD has been able to become more effective and efficient in controlling this vector of great concern.

### 2.3. Truck Traps

Truck-mounted traps have long been used to collect large numbers of mosquitoes in-flight. Chamberlain and Lawson [85] were the first to investigate the use of a vehicle-mounted trap for sampling insects in flight. Bidlingmayer [86] first used a truck-mounted trap to study midges, *Culicoides* sp. Latreille (Diptera: Ceratopogonidae), then slightly modified it for mosquitoes [87]. Carroll and Bourg [88] were able to determine peak flight times for several species of interest by employing a truck trap driven at fixed time intervals. The FKMCD constructed a truck trap and used it to determine diel flight period of *A. taeniorhynchus*. Pruszynski [89] reported that the truck trap collected no less than five species of mosquitoes and that the species of interest, *A. taeniorhynchus*, was flying in greatest numbers between one-half hour and three hours post-sunset. This information allowed FKMCD to adjust its spray truck schedule to ensure that more ground-based treatments were made when mosquitoes were flying in the greatest numbers.

Truck traps have an advantage over light traps. Whereas light traps compete with a natural light source (the moon), truck traps do not confound light sources to attract insects [90]. There are limitations to using truck traps. Truck traps are useful only where pickup trucks can be driven, limiting their use to roadways or relatively flat terrain. They cannot be used to sample marshy or wooded areas unless there is a drivable roadway through the area. This can bias the species composition of the collection [88]. Furthermore, meteorological conditions can impact the number and perhaps species composition of mosquitoes collected in truck traps [90,91]. Whereas other trapping methods collect mosquitoes at a point source, truck traps collect them along a transect, or a loop, which may make interpretation of catch problematic [91].

### 2.4. Gravid Traps and Oviposition Traps

Oviposition traps exploit the egg-laying behavior of female mosquitoes and can be used for both surveillance and control [92]. The gravid trap developed by Reiter [93] is used occasionally by FKMCD; this trap was used during surveillance for West Nile virus [52]. Although an infusion made from Bermuda grass is a highly attractive additive to gravid traps [94,95]), FKMCD used horse manure, collected from the Monroe County Sheriff’s Office Animal Farm, as an attractant.

Buxton and Hopkins [96] were the first to use artificial oviposition traps for collecting *A. aegypti*. Oviposition traps are easily made and are used to quickly gauge *A. aegypti* population levels [97]. The FKMCD used oviposition traps as a surveillance method to determine numbers of female mosquitoes in a field trial of pyriproxifen [98]. Oviposition traps were used in conjunction with BG Sentinel traps to measure efficacy of wide-area liquid larviciding for control of *A. aegypti* in Key West, FL [73]. The FKMCD also used oviposition traps to monitor and verify the establishment of *A. albopictus* in the Florida Keys [36] and to obtain eggs for bioassays during an investigation of mechanisms of resistance to pesticides by *A. aegypti* [99].

### 2.5. Landing Rate Counts

The Landing Rate Count (LRC) is simply a total number of mosquitoes landing on an observer within a defined time period [100]. The first reported use of human landing rate counts was by Headlee [101] working in New Jersey, USA. The FKMCD uses landing rate counts both to assess the need for adulticide treatments and the success or failure of larvicide and adulticide treatments.

The FKMCD uses LRCs to gauge the relative abundance of adult *A*. *taeniorhynchus* mosquitoes, which is the most abundant mosquito collected in the Florida Keys [27]. *Aedes taeniorhynchus* is not a vector of human pathogens, but precautions such as long sleeves and pants are worn to prevent actual bites from this species. Landing rate counts are not conducted to measure abundance of *A. aegypti* nor other potential mosquito vector species. Sixteen FKMCD inspectors maintain approximately 266 landing rate count sites throughout the FKMCD jurisdiction. At the beginning of each work day, an inspector will spend more than two hours measuring LRC at assigned sites. The inspector may begin the LRC by first brushing against some nearby bushes where adult female mosquitos may be resting. Then, they stand with legs hip-width apart and arms in front or outstretched and begin counting for one minute the number of mosquitoes that land on the front of their bodies, regardless of species. Those data are then recorded into the FieldSeeker^®^ (Frontier Precision, Bismarck, ND, USA) database and are used to determine relative host-seeking mosquito abundance in various areas. The data inform operations staff whether to initiate adulticidal control.

The LRC is scientifically inaccurate, and can have many confounding factors. While FKMCD inspectors attempt to control the LRC metric to be as consistently accurate as possible, it is inevitable that deviations occur. Some days, inspectors must delay or cancel a LRC due to weather or other matters including time off. In those cases, a LRC may not be measured at an ideal time for mosquito activity or another inspector may take their place which alters uniformity. Mosquitoes have been shown to be attracted to some people more than others [102]. Mosquitoes may land on a part of the inspector and go unseen. There may be too many mosquitoes to count in one minute. Mosquitoes may alight but not land. In sum, LRCs are subjective to who and when the count is performed.

There is no collection method that is better for attracting human-biting mosquitoes than human bait catches [103]. Although studies using LRCs have provided invaluable information that would have been impossible to obtain otherwise [104], and there are no state rules or legislation regulating the use of LRCs in Florida, there are ethical considerations using human subjects to attract host-seeking mosquitoes; some may be vectors of disease organisms [19,63,105,106]. Whereas in malaria research there appears to be little risk to persons doing human bait catches (provided they have proper prophylaxis) [107], with the potential transmission of viral pathogens this risk is unacceptable. It is imperative to prevent harm to come to persons serving as human bait during LRCs [108]. For this reason, FKMCD is investigating methods to replace human LRCs, like the BG Counter (Biogents AG, Regensburg, Germany).

The BG Counter is an autonomous mosquito trap that differentiates mosquitoes from other insects, counts them, and wirelessly transmits the information to a webpage. From the webpage, operators can program the trap to collect for mosquitoes at specific time intervals in 15-min increments. The release of carbon dioxide as an attractant and a ventilator fan attached to a BG-Pro trap (Biogents AG, Regensburg, Germany) draws the insect in through the Counter that classifies an insect by size when it breaks an infrared light barrier [109,110]. The traps are powered by either A/C current or a 12 V battery charged by solar panel. Solar power enables the trap to operate in more remote locations. However, cellular signal is necessary for data transmission.

The FKMCD has been evaluating the BG Counter as a replacement for human LRCs for multiple reasons. As mentioned above, LRCs are ethically questionable by enabling a small, but existent risk of possible vector-borne disease transmission. LRCs can vary from the time they are taken during the day and from one inspector to another. The BG Counter allows a standardized approach to gathering relative mosquito population data as all traps can be programed to collect mosquitoes at the exact same time using the exact same methods. This consistency will help the District make operational decisions more decisively. Lastly, BG Counters are a time-saving measure for many inspectors as they will no longer dedicate two hours each day to driving to distant and isolated LRC sites. Instead, they will have more time to devote to surveying for possible larval habitats and treating for mosquitoes. While evaluations from earlier versions showed varying accuracy based on trap counts [111], evaluations conducted by FKMCD on the most recent model showed the BG Counter to be 93% accurate in identifying mosquitoes verses other non-mosquito species [110] with other groups obtaining similar results [109,112]. The District continues to evaluate this product and is methodically integrating BG Counters into current LRC sites to establish baseline data with the eventual goal of eliminating most human LRC sites.

## 3. Larval and Pupal Surveillance

Larval surveillance has been the historical bedrock of mosquito surveillance and abatement since before the discovery that mosquitoes transmit disease. The earliest published larviciding methods were oil based and treating the breeding sources was the only way of reducing mosquito numbers [113,114]. Since that time, mosquito abatement has evolved with new technologies and increased understanding of mosquito biology and behavior. Efficient integrated pest management practices now rely on visiting mosquito larval habitats to determine species and life stages. Findings in the field dictate which control methods are most effective in order to reduce the number of pestiferous and vector mosquitoes. This is the tenet of FKMCD’s larval and pupal surveillance program.

At FKMCD, larval and pupal surveillance are generally regarded together and surveillance takes either a field or domestic approach. In field surveillance, inspectors dip for larvae in mangrove swamps, woodland pools, solution holes, grassy low areas and many other field sites throughout the Keys. The most pestiferous floodwater species found in the Keys is typically *A. taeniorhynchus*. Domestic surveillance at FKMCD, incorporates the recognition and treatment of any artificial container at private homes, businesses, and public land. Larval habitats found during domestic inspections can be any artificial or natural water-holding container ranging from cryptic (i.e., decorative conch shells, plastic bottle caps, etc.) to obvious (i.e., concrete storm drains, ornamental ponds, etc.). The target of domestic larval surveillance is *A. aegypti*, the primary vector of mosquito-borne disease in the Florida Keys. Both field and domestic larval surveillance are important in preventing adult biting mosquitoes for both disease prevention and comfort of the public.

Each FKMCD inspector is assigned a geographic area that includes both field and domestic sites. Most assigned areas are demarcated either by the end of islands or split up by mile markers (MM) depending on the size of the islands (i.e., Grassy Key–Conch Key, MM 98.5–MM 102.5). Each inspector’s assigned area is considered their responsibility and they are expected to visit field sites regularly or as necessary following tide or rain events. This consistent observation of the same area allows the inspectors to familiarize themselves with each field site and gauge how tidal incursion and rainfall will affect each unique site. Due to frequent visitations to field sites, especially during the wet season, inspectors know when and how much these sites can flood from previous visits. This observational knowledge is a useful indicator when dipping for floodwater species such as *A. taeniorhynchus*. Oviposition preference of *A. taeniorhynchus* in South Florida at times may seem non-existent, as emerging adults seem to emanate from every recently flooded water source. However, oviposition preference may rely on existing detritus material, lack of predators, and frequency of tidal flooding [115,116]. The FKMCD inspectors are taught to dip for larvae at sites they suspect or know to harbor flood water species, with an emphasis on shaded areas, near the water’s edge, or between the previous water mark and the current higher water mark. These areas are typically where floodwater mosquito larvae are likely to have hatched, and risen to the surface. Dipping protocol at FKMCD is situationally dependent as variety of conditions and target species exist in both field and domestic sites. O’Malley [117] succinctly detailed the most commonly used techniques for dipping field sites in “Seven Ways to a Successful Dipping Career” and these techniques are taught to field inspectors at FKMCD and used at their discretion.

Discovery of mosquito larvae in field sites provides the basis for aerial Bti treatment. Inspectors must be adept at determining instar of field caught mosquito larvae, because the Florida Keys typically have ideal larval development temperatures [118]. When inspectors cannot reach larval habitats quickly enough, larval development reaches a non-feeding 4th instar or the pupal stage before observation and treatment. At this stage, Bti is not an effective treatment so monomolecular films are necessary to treat field sites. Speed and efficiency is of the utmost importance at sites such as the National Key Deer Refuge, as federally protected land and some state managed lands restrict the use of larvicides other than Bti. Although inspectors are trained in determining the genus and stage of larval development of field collected specimens, accurate species identification can only take place under a microscope at FKMCD facilities.

Domestic larval surveillance is primarily performed by the FKMCD field inspectors using a variety of sampling tools including dippers (Clarke, St. Charles, IL, USA), larval trays (BioQuip, Rancho Dominguez, CA, USA), 45 mL turkey basters (Publix brand, Lakeland, FL, USA), or sweeping through storm drains in an increasingly deeper “S” shaped movement using aquarium nets affixed to a PVC pipe [119,120]. In Key West some inspectors focus solely on domestic or storm drain inspections in neighborhoods. These inspectors become familiarized with homeowners and business owners. Familiarity with the public offers inspectors continued access, opportunities for education on source reduction, and the ability to monitor and treat larval habitats that the owners are unable or unwilling to remove. Extreme situations may require involvement of city or county Code Compliance offices.

Once larvae and/or pupae are collected, samples are brought from the field to the laboratory in 6 oz. straight walled polystyrene jars (US Plastics, Lima, OH, USA), 70/400 lid). All sample collections are considered a representative sample of the total field population. Samples brought into the laboratory are identified as soon as possible by a certified mosquito identification specialist. Species, dominant instar, and number of larvae and pupae are recorded in FieldSeeker for each sample. Larval samples of *A. aegypti* are collected by inspectors from every fifth house visited starting with the first house where larvae are found. The exception to this guideline occurs when there is an active mosquito-borne disease transmission when all live *A. aegypti* larvae found are sampled and returned to the laboratory. As such, during the 2020 dengue outbreak in the Upper Keys, every home that had *A. aegypti* larvae was sampled when possible. During this time and total of 767 samples were collected totaling 12,705 *A. aegypti* larvae and 1337 *A. aegypti* pupae [121]. Each sample was processed and data were recorded on the same day of collection. These real time data provided FKMCD operations staff with a basis to determine which areas required treatment with the Bti product VectoBac WDG (water dispersible granule) using an A1 Super Duty Mist Sprayer (A1 Mist Sprayers, Ponca, NE, USA) or by helicopter for area wide treatments [73,122]. Follow up inspections of VectoBac WDG treated areas revealed in situ larval mortality observed by inspectors.

Detailed surveillance occurs when field collected specimens are placed into a sample jar and returned to the laboratory. Data are recorded in FieldSeeker, a data management system that uses geographic information system (GIS) software to map field and domestic sites and save historical information for each field site or address. Historical inspection data and operational notes on each site assists inspectors in remembering any cryptic habitats that may easily be overlooked during subsequent inspections. Geographical Information Systems such as FieldSeeker are useful for operational response during disease transmission cycles as seen during the Key Largo dengue outbreak in 2020. This data management system allows FKMCD personnel to record the species, quantity of each species present, type of habitat where the larvae were found, where the sample was taken, and if any chemical was used to treat the site. In previous larval surveys of the Florida Keys [123,124], the authors discussed larval indices and container habitats utilized by mosquito species in the Keys. These studies calculated indices long after the data had been collected and could not be used for operational purposes. Presently, by using FieldSeeker, larval indices can be calculated in real time in order to refocus inspector efforts when necessary, A color-coded indicator for each address shows which homes have not been visited in the past 30 days.

Larval surveillance can also be used as an early detection method and establishment indicator for invasive species. With the introduction of *A. albopictus* into Florida [125] and its ability to transmit arboviruses [126,127,128], the necessity to discern *A. aegypti* from *A. albopictus* in the larval stage was apparent. *Aedes albopictus* has a slightly larger flight range than that of *A. aegypti*, less need to cohabitate with humans, and the ability to outcompete *A. aegypti* in a majority of mainland Florida [129,130,131]. In 1993, *A. albopictus* was first discovered in its larval form in the Ocean Reef Club in northern Key Largo [36]. At FKMCD, operational larval sampling started in the year 1998 and has since become standard operations for the domestic inspection program. Since its inception, the larval inspection program helped determine that *A. albopictus* had since established on 30 islands in the Florida Keys [36]. Murray et al. [36] identified 441 larval samples of *A. albopictus* collected by inspectors. Only 101 positive ovicups and 49 adult specimens were recorded in the same time period. Although it is more time and labor intensive, domestic larval surveillance has provided more data points than either adult or ovicup surveillance. Larval surveillance has also provided FKMCD with several species records of *Toxorhynchites rutilus rutilus* (Coquillett), a non-blood feeding mosquito unlikely to be collected by CO2-baited traps used by FKMCD for adult mosquito trapping and surveillance [132]. Despite criticisms of larval surveys [133,134], FKMCD has a robust domestic program that affords inspectors the chance to remove larval habitats, treat larvae, map out larval densities, and determine further treatment actions based on the larval surveillance they perform daily. In doing so, this prevents adults from causing discomfort or spreading mosquito-borne illness.

## 4. Insecticide Resistance Monitoring

Vector control programs have used chemical insecticides to control larval and adult mosquito populations for decades in order to keep nuisance species under control and to interrupt virus transmission. The overuse of insecticides over time has led to resistance of these chemicals in mosquito populations [135]. Physiological mechanisms in mosquitoes can work to decrease the effect of the insecticide leading to resistance. Mechanisms can be biochemical (e.g., target site modifications and metabolic resistance) or morphological (e.g., cuticular resistance) [136]. Secondary behavioral adaptations can develop in mosquitoes when physiological mechanisms allow them to recognize the pesticide as harmful (e.g., avoidance, limiting exposure, and compensation) [137]. The FKMCD regularly monitors for insecticide resistance in both larval and adult abatement products to ensure treatment effectiveness.

### 4.1. Adulticides

There are only two classes of insecticides currently available for the control of adult mosquitoes; organophosphates and pyrethroids. Of these two insecticide classes, pyrethroids are considered a more environmentally friendly option by the general public and provide a rapid knockdown [138]. The continued favor of pyrethroid insecticides and the previous use of DDT, both of which have the same mode of action, has led to resistance in some mosquito populations [138]. Due to the increase in pyrethroid resistance observed in mosquitoes, the Centers for Disease Control and Prevention (CDC) has initiated programs to conduct resistance surveillance in the US. Resistance surveillance can provide mosquito control agencies with important information for application decisions under standard operating conditions as well as during a mosquito-borne disease outbreak. A relatively simple way to look at resistance in mosquito populations is the CDC bottle bioassay. This surveillance tool provides insight into whether active ingredients (AI) in the pesticides used against mosquito populations are effective or if the mosquitoes are becoming resistant to the product. The bottle bioassay works by coating the inside of glass bottles with a known amount of insecticide and then enclosing adult mosquitoes in the bottles. Mosquitoes are observed for two hours in the bottles with checks at 5, 10, and every 15 min thereafter to determine how many are dead. The mortality rate observed is compared to a pre-determined threshold or a susceptible population run concurrently to determine if resistance is likely present in the population. If 97–100% mortality is observed at the diagnostic time, the bottle bioassay indicates that the mosquito population is susceptible to the insecticide. A mortality rate of 90–96% indicates the mosquito population is developing resistance and <90% mortality at the diagnostic time implies resistance [139].

In the Florida Keys, insecticides are part of FKMCD’s integrated mosquito management program. Bioassays on these insecticides have been conducted by the District as far back as 1999 on both formulated and technical-grade active ingredients. If resistance is detected in any of the insecticides used by FKMCD, field trials are conducted to determine efficacy of the product. The adulticides tested in bottle bioassays by FKMCD include both pyrethroid and organophosphate products. Ultra-low volume (ULV) treatments by truck have included the following permethrin products: Biomist^®^ 30+30 ULV (Clarke Mosquito Control Products Inc., Roselle, IL, USA), Evoluer^®^ 30-30 ULV (Value Garden Supply, St. Joseph, MO, USA), Kontrol^®^ 30-30 (Univar Environmental Sciences, Austin, TX, USA), and Permanone^®^ 30-30 (Bayer, Research Triangle Park, NC, USA). Handheld foggers are used by FKMCD to apply Duet^®^ (AI: prallethrin and sumethrin) (Clarke Mosquito Control Products Inc., Roselle, IL, USA) during household inspections for *A. aegypti*. All five of these pyrethroid products also contain the inhibitor piperonyl butoxide (PBO). PBO is a pesticide synergist, inhibiting the insect’s natural defense mechanisms, allowing the pesticide to work more effectively [140]. For barrier treatments, Wisdom TC Flowable (AI: bifenthrin) (AMVAC Chemical Corporation, Los Angeles, CA, USA) is applied by truck, all-terrain vehicle (ATV), and backpack sprayer. Fyfanon^®^ ULV and Fyfanon EW (AI: malathion) (FMC, Philadelphia, PA, USA) are organophosphate products rotated into ULV truck applications. Dibrom^®^ (AI: naled) (AMVAC Chemical Corporation, Newport Beach, CA, USA) is an organophosphate applied aerially by FKMCD.

In response to the 2009 and 2010 dengue outbreak in Key West, FL, a study was conducted to determine the efficacy of aerial application of Dibrom against the *A. aegypti* population in Key West, FL. A bottle bioassay was conducted in the FKMCD laboratory to determine if *A. aegypti* from Key West were susceptible to the organophosphate insecticide Dibrom (naled). Results were 100% mortality in 30 min, implying susceptibility. A field trial was conducted and indicated that Dibrom can work against *A. aegypti* in open spaces (73% mortality) but is unlikely to control *A. aegypti* resting in cool, shady areas (41.3% mortality). This field trial provided evidence that even when mosquitoes are susceptible to an active ingredient in a bottle bioassay, it does not necessarily mean good control in the field. Since limited success was observed using Dibrom to control *A. aegypti* in Key West, other methods of control should be used during an outbreak to control the population (unpublished internal document).

In addition to adulticide products currently used by FKMCD, new active ingredients are evaluated as they become available. In 2019, bottle bioassays were conducted to test the toxicity of tau-fluvalinate, lambda-cyhalothrin, and alpha-cypermethrin (Sigma-Aldrich, St. Louis, MO, USA) against *A. aegypti* adults. Serial dilutions resulted in an LD50 = 32.22 ppm for tau-fluvalinate, LD50 = 0.91 ppm for lambda-cyhalothrin, and LD50 ≤ 0.001 for alpha-cypermethrin. Alpha-cypermethrin had 100% mortality against local *A. aegypti* adults with the lowest toxicity among the three options [141]. Knowing that alpha-cypermethrin had such positive results against our local *A. aegypti* population, FKMCD can prioritize testing new products with this active ingredient in the future.

While mosquitoes are the District’s primary focus with regard to insecticide resistance, FKMCD has also had the opportunity to test the effects of mosquito control products on some non-targets. In 2016, FKMCD collaborated with the United States Department of Agriculture (USDA) during a screwworm fly, *Cochliomyia hominivorax* (Coquerel) (Diptera: Calliphoridae), outbreak on Big Pine Key, FL. An investigation was conducted to determine whether mosquito control adulticides would have a negative impact on released sterile screwworm flies. Bottle bioassays were completed using the three main adulticides applied by FKMCD; permethrin (43 µg) was most toxic of the three to the sterile screwworm flies provided by USDA, then malathion (474.56 µg), and the least toxic was naled (25 µg). These doses are the same used by FKMCD to evaluate resistance in mosquitoes. Dosage tests were also conducted to determine the concentration required to kill screwworm flies with the product Evoluer 30-30 (AI: permethrin). A dose as low as 4.3 µg was enough to kill 50% of the flies. Through outdoor cage trials, it was determined that screwworm flies were more susceptible to permethrin than local salt marsh mosquitoes, *A. taeniorhynchus*, but less susceptible to both organophosphate active ingredients, malathion and naled. However, organophosphate applications may be preferred after a recent sterile insect release if a mosquito-borne disease outbreak occurs during this time [142].

In addition to working with sterile screwworm flies with the USDA, FKMCD collaborated with a variety of stakeholders to study non-target effects on larvae of an endangered butterfly. Larvae of the Miami blue butterfly, *Cyclargus thomasi bethunebakeri* (Comstock and Huntington) (Lepidoptera: Lycaenidae), and caged *A. taeniorhynchus* adults were placed in the field and naled was applied aerially at a rate of 54.8 mL per hectare. In all six field trials, naled was effective at killing caged *A. taeniorhynchus* adults. The mean concentration of naled in the designated spray zone (811.7 µg/m^2^) resulted in approximately 6% mortality of Miami blue butterfly larvae. Increasing mortality was observed in areas where naled concentrations reached 1000 µg/m^2^, occurring where wind moved the product unevenly across the landscape. It is important to understand how wind speed and direction, aircraft speed and altitude, and landscape design affects the movement of naled across the spray zone to better reduce naled residue intensification in some areas. Concentrations in the nearby drift zone suggest that there is very low risk of mortality for Miami blue butterflies outside of the designated spray zone [143].

The District has participated in a number of side studies but one of the main targets is to keep track of resistance in the mosquito populations in the Keys. A collaboration between FKMCD and the CDC examined not only whether resistance was present in the *A. aegypti* populations of the Florida Keys, they also looked specifically at what mechanisms were involved in any resistance observed. The FKMCD conducted bottle bioassays on *A. aegypti* from Key West and Vaca Key against Biomist 30-30 and detected resistance. Biomist 30-30 is a formulated product composed of permethrin (30%), piperonyl butoxide (PBO) (30%), and other ingredients (40%). The CDC conducted further bottle bioassays and found *A. aegypti* collected from Key West to be susceptible to permethrin, suggesting that PBO may have an unidentified role in resistance. Mosquitoes tested by the CDC were susceptible to malathion and building resistance to bifenthrin. Bifenthrin was tested in conjunction with the enzyme inhibitors PBO, diethyl maleate (DEM), and S.S.S.-tributylphosphorothioate (DEF) and 100% mortality was observed at 75, 15, and 60 min respectively. Resistance to bifenthrin was reduced with the addition of DEM and DEF suggesting that glutathione transferase and esterase are involved with resistance. The presence of detoxifying enzymes was detected at multiple locations in the Florida Keys including Key Largo, Upper Matecumbe, Vaca Key, Stock Island, and Key West. Each location had different levels and mechanisms present. Key Largo had the highest levels of all enzymes tested, followed by Upper Matecumbe Key and Key West. Vaca Key had the lowest levels detected. This shows the importance of conducting resistance testing by island for short-ranged mosquito populations, such as with *A. aegypti*, and not just sampling one location for the entire county [99]. For this reason, testing for resistance by island throughout the Florida Keys is part of the District’s 5-Year Strategic Plan. The resistance status of *A. aegypti* populations is important for pesticide selection to effectively respond to outbreaks such as those of dengue, chikungunya, and Zika. Knowing what specific mechanism is used by the mosquito to gain resistance can help in selection of proper control methods.

### 4.2. Larvicides

Larvicides are indisputably the most important abatement products for mosquito control, and FKMCD relies heavily on Bti for treating *A. taeniorhynchus* in field sites. This is the only active ingredient available for use on some large holdings in the Florida Keys, including the Florida Keys Wildlife Refuges Complex. VectoBac^®^ G (active ingredient: Bti) (Valent BioSciences Corporation, Libertyville, IL, USA) is applied by hand, VectoBac GS is applied by helicopter, and VectoBac WDG is applied by helicopter, truck, and backpack sprayer. VectoLex^®^ FG (active ingredient: *Lysinibacillus sphaericus* Ahmed et al. (Bacillales: Bacillaceae) is used in coordination with VectoBac G for treating sites with high numbers of *C. quinquefasciatus* larvae such as drains and sewage treatment plants. Other larvicides are used for FKMCD’s domestic program against *A. aegypti* and *Cx*. *quinquefasciatus*, including: mosquitofish (*Gambusia* spp., Cyprinodontiformes: Poeciliidae), Altosid^®^ XR, Altosid pellets, Altosid XR-G (AI: methoprene) (Wellmark International, Schaumburg, IL, USA), and Natular^®^ DT, Natular G, Natular G30, Natular XRT (AI: spinosad) (Clarke Mosquito Control Products Inc., Roselle, IL, USA). Mineral oils are also used including: Kontrol (Univar Environmental Sciences, Austin, TX, USA), Cocobear™ (Clarke Mosquito Control Products Inc., Roselle, IL, USA), and BVA oil (BVA Oils, New Hudson, MI, USA).

Larval bioassays are conducted at FKMCD in response to treatment failure. When a field inspector notices a product not working as expected, and human error is ruled out, the research department conducts laboratory assays to determine if resistance is the problem. Currently, there is no standardized larvicide lab bioassay methodology provided by the CDC. However, there are resources available from the Pacific Southwest Regional Center of Excellence in Vector-Borne Diseases which is supported through a cooperative agreement between the CDC and UC Davis [144].

In 2017, field inspectors noticed an efficacy problem in some of the storm drains in Marathon, FL when treating with Natular XRT tablets, specifically with *Cx*. *quinquefasciatus* larvae. Larval bioassays of local *C*. *quinquefasciatus* collected from the problem drains and a susceptible strain of *C*. *quinquefasciatus* from USDA Center for Medical, Agriculture, and Veterinary Entomology laboratory in Gainesville, FL, were conducted to examine resistance against the active ingredient spinosad. Within 24 h of exposure, 100% mortality was observed for the USDA susceptible strain and 100% mortality was observed for the field-collected larvae within 48 h of exposure [145]. After resistance was ruled out, field trials were conducted to determine if Natular XRT tablets provide effective control in storm drains in the Middle Keys. On week 7, control started to decline and a dark green film was observed on the tablets. This film was likely hindering the dispersion of the product through the water column. Water samples were collected from the storm drains and tested to determine any differences in water quality between test and control drains. Water samples revealed a significant difference in alkalinity and water hardness between test and control drains. Since solubility of spinosyns in water is known to decrease as pH increases, this may be an important factor to consider when selecting field sites for treatment with this active ingredient [146]. It was determined that the environmental circumstances for these particular drains require an alternate control method [145].

In 2001, laboratory bioassays were used to examine possible methoprene resistance in *A. taeniorhynchus* from No Name Key, FL. Larvae were collected from the field on No Name Key and shipped overnight to the Public Health Entomology Research and Education Center (PHEREC). The larvae were reared to adults, blood-fed, and eggs were collected. The F1 generation was used at 3rd-instar for larval bioassays. A susceptible population of *A. taeniorhynchus* was also tested as a comparison. The bioassay showed no significant difference in tolerance of methoprene between the two populations [147].

More recently, methoprene resistance was measured in three different strains of *A. aegypti* collected from Key West, Marathon, and Key Largo along with a methoprene-susceptible strain. The LD50 for each strain was 5.103 ppm, 5.7 ppm, 12.2 ppm and 0.264 ppm for Key West, Marathon, Key Largo, and the susceptible strain, respectively [148]. This again confirms that location strains can have varying degrees of tolerance and that it is important to establish baseline information to monitor changes to resistance.

Baseline data were also collected on the toxicity of naled and eugenol against *A. aegypti* larvae collected from Marathon, FL, in 2018. Second-instar larvae were added to dilutions of 1–400 ppm of eugenol (Sigma-Aldrich, St. Louis, MO, USA) and 0.1–2 ppm of naled (AMVAC Chemical Corporation, Los Angeles, CA). The bioassays determined an LD50 of 24.92 ppm for eugenol and an LD50 of 0.55 ppm for naled. This test provides a baseline for treatment application and future resistance testing conducted by the District [149]. It is a priority of FKMCD to search out and identify additional products for use in response to mosquito-borne disease outbreaks. By rotating in additional pesticide options for treatment, FKMCD may be able to reduce the extent of resistance for more preferred products.

## 5. Conclusions

Before operational decisions can be made by mosquito control programs, a comprehensive mosquito monitoring program must be in place. Species composition, relative abundance, and seasonal distribution of the local mosquito fauna need to be known and constantly watched in order that operations can respond to changes in numbers of pestiferous mosquitoes or increases in vector species. Ideally, adults, pupae, larvae, and eggs should be routinely collected and identified and the dynamics of the local mosquito community tracked over time. Periodic bioassays should be conducted to detect insecticide resistance prior to it becoming a problem. Results of such studies should be written up and submitted for publication in order that documentation of investigations is available for future use.

## Figures and Tables

**Table 1:** Type and number of traps set weekly and Landing Rate Counts (LRC) per day for routine surveillance in 3 FKMCD subdivisions of the Florida Keys, 2021. (ABC, American Biophysics Company; CDC, Centrs for Disease Control; BG, Biogents).

	Trap Type			
	ABC/CDC	BG Sentinel	Oviposition	LRC (Per Day)
Lower Keys	33	18	6	121
Middle Keys	10	3	0	46
Upper Keys	19	11	0	56

## Data Availability

Not applicable.
